# Cognitive Change during the Life Course and Leukocyte Telomere Length in Late Middle-Aged Men

**DOI:** 10.3389/fnagi.2016.00300

**Published:** 2016-12-09

**Authors:** Lene Rask, Laila Bendix, Maria Harbo, Birgitte Fagerlund, Erik L. Mortensen, Martin J. Lauritzen, Merete Osler

**Affiliations:** ^1^Department of Neuroscience and Pharmacology, University of CopenhagenCopenhagen, Denmark; ^2^Department of Clinical Neurophysiology, Rigshospitalet - GlostrupGlostrup, Denmark; ^3^Center for Healthy Aging, University of CopenhagenCopenhagen, Denmark; ^4^Pain Research Group, Department of Anaesthesiology and Intensive Care Medicine, Odense University HospitalOdense, Denmark; ^5^Department of Clinical Genetics, Vejle HospitalVejle, Denmark; ^6^Center for Neuropsychiatric Schizophrenia Research and Lundbeck Foundation Centre for Clinical Intervention and Neuropsychiatric Schizophrenia Research, University of Copenhagen, Psychiatric Centre GlostrupGlostrup, Denmark; ^7^Department of Public Health, University of CopenhagenCopenhagen, Denmark; ^8^Research Center for Prevention and Health, Rigshospitalet - GlostrupGlostrup, Denmark

**Keywords:** cognitive function, telomere length, mental health, birth cohort study, aging

## Abstract

**Importance:** Cognitive skills are known to decline through the lifespan with large individual differences. The molecular mechanisms for this decline are incompletely understood. Although leukocyte telomere length provides an index of cellular age that predicts the incidence of age-related diseases, it is unclear whether there is an association between cognitive decline and leukocyte telomere length.

**Objective:** To examine the association between changes in cognitive function during adult life and leukocyte telomere length after adjusting for confounding factors such as education, mental health and life style.

**Design, Setting, and Participants:** Two groups of men with negative (*n* = 97) and positive (*n* = 93) change in cognitive performance were selected from a birth cohort of 1985 Danish men born in 1953. Cognitive performance of each individual was assessed at age ~20 and 56 years. Leukocyte telomere length at age ~58 was measured using qPCR. Linear regression models were used to investigate the association between cognitive function and leukocyte telomere length.

**Results:** Men with negative change in cognitive performance during adult life had significantly shorter mean leukocyte telomere length than men with positive change in cognitive performance (unadjusted difference β = −0.09, 95% CI −0.16 to −0.02, *p* = 0.02). This association remained significant after adjusting for smoking, alcohol consumption, leisure time activity, body mass index (BMI) and cholesterol (adjusted difference β = −0.09, 95% CI −0.17 to −0.01, *p* = 0.02) but was non-significant after adjusting for smoking, alcohol consumption, leisure time activity, BMI, cholesterol, current cognitive function, depression and education (adjusted difference β = −0.07, 95% CI −0.16 to −0.01, *p* = 0.08).

**Conclusion and Relevance:** Preclinical cognitive changes may be associated with leukocyte telomere length.

## Introduction

Cognitive performance seems to decline during adult life under the influence of genetic and environmental factors (Deary et al., 2009; Tucker-Drob and Briley, 2014). It has been suggested that cognitive decline is caused by a self-perpetuating cycle of oxidative stress triggering neuroinflammation, which further promotes oxidative stress as well as the suggested underlying neurodegeneration and cell death (Cai et al., 2013). Recently, cognitive decline was associated with leukocyte telomere length (LTL) and the LTL is assumed to reflect the accumulated burden of inflammation and oxidative stress occurring during the life course (Eitan et al., 2014). This relationship may therefore be influenced by same mechanisms such as oxidative stress and inflammation but the association may also be bi-directional.

In a number of cross-sectional studies (outlined in Supplementary Table 1), mainly among adults above 60 years, short LTL has been associated with cognitive impairment (Valdes et al., 2010; Der et al., 2012; Ma et al., 2013). Other studies have found no correlation between LTL and cognitive function (Harris et al., 2006; Bendix et al., 2011, 2014a; Cohen-Manheim et al., 2016). Furthermore, two cohort studies have found that short LTL predicts cognitive decline (Martin-Ruiz et al., 2006; Yaffe et al., 2011). In the Nurses' Health Study, short LTL in women above age 70 was modestly associated with subsequent cognitive decline (Devore et al., 2011), and in 70–79 year old adults from the Health ABC study short LTL predicted a 7 year decline in Mini Mental Test score (Yaffe et al., 2011). In two cohort studies, however, cognitive decline did not predict subsequent differences in LTL. In 70-year olds from the Lothian 1936 birth cohort, LTL at age 70 was not associated with cognitive changes occurring between ages 11 and 70 (Harris et al., 2012), nor was there any association between cognitive decline and LTL in an Australian population-based study (Mather et al., 2010).

The previous cohort studies have examined the relationship between LTL and cognitive decline in follow-ups of 2–7 years (Martin-Ruiz et al., 2006; Mather et al., 2010; Devore et al., 2011; Yaffe et al., 2011), mostly in persons above 70 years (Martin-Ruiz et al., 2006; Devore et al., 2011; Yaffe et al., 2011; Harris et al., 2012) (Supplementary Table 1). Thus, it remains uncertain as to whether or not LTL is a marker of cognitive decline in early aging.

The aim of the present study was to examine the association of change in cognitive function occurring between age ~20 and 56 with LTL measured at age ~58, as well as to investigate the extent to which potentially confounding factors: smoking, alcohol consumption, leisure time activity, Body Mass Index (BMI), serum cholesterol, indicators of mental function and education might influence any such association.

## Materials and methods

### Study population

Participants were recruited from the Metropolit 1953 Danish male birth cohort, which includes 11,532 men born in 1953 in Copenhagen (Osler et al., 2009). These men were all cognitively assessed at age ~20 and again in 2009–10 at age ~56 as part of the Copenhagen Aging and Midlife Biobank project (CAMB) (Avlund et al., 2014; Hansen et al., 2014). Among the 1985 members of the Metropolit cohort who had participated in both cognitive tests, a linear regression analysis was carried out with the cognitive test score at age ~20 as single predictor and the cognitive test score at age ~56 as outcome. Each individual's standardized residual from the regression line was considered a measure of the relative cognitive change between the two tests for that particular individual. To ensure sufficient variance in the magnitude of cognitive change, we invited the participants with the largest positive residuals [termed group 1 (residual range 0.96–2.80)] and the largest negative residuals [termed group 2 (residual range −2.83 to −0.99)] to participate in the study. Outliers defined as individuals with standardized residuals larger than 3 were excluded. In total, 552 participants were invited by letter to participate in a neurophysiological examination and blood sampling (249 in group 1; 303 in group 2). Based on participant interviews 36 persons were excluded (abuse of alcohol or drugs, certain neurological disorders, certain neurological, mental or psychiatric diagnoses, certain depressive diseases, former traumatic brain injury or contraindication of MR scanning). Of the remaining participants, 207 (102 in group 1; 105 in group 2) chose to participate in the examination period from 2010 to 2013 (~58 years). LTL was not measured for 13 participants (7 and 6 from group 1 and 2, respectively) (due to lack of blood material or unknown reasons). One included participant had a persistent depression (group 2). Within-group comparisons of residuals showed no differences between included participants, participants who were invited but did not want to participate, and those who were excluded. The inclusion procedure is described in detail in previous publications (e.g., Hansen et al., 2014).

The study was approved by the local ethical committee (De Videnskabsetiske Komiteer for Region Hovedstaden). All participants provided written informed consent. The study has also been registered by the Danish Data Protection Agency.

### Measurements

#### Cognitive change

At age ~20 years the participants completed the Danish draft board intelligence test Børge Prien's Prøve (BPP) as a part of the draft board assessment program. The 45-min BPP test comprises letter matrices, verbal analogies, number series, and geometric figures. The score, which is the total number of correct answers out of 78 questions, has proved to correlate to 0.82 with the Wechsler Adult Intelligence Scale (Teasdale, 2009). In the CAMB follow-up examination in 2009/2010 participants completed a short version of the Intellegenz-Struktur-test 2000R (IST), which matches the BPP test and comprises verbal analogies, number series, and sentence completion (Osler et al., 2013; Mortensen et al., 2014). Relative cognitive change was assessed by standardized residual from regression analyses on the two tests as described above in the section “Study population.”

#### Leukocyte telomere length (LTL)

Non-fasting blood samples were collected during the examination day. Buffy coat was prepared from whole blood and DNA was purified using Maxwell system for automated extraction from buffy coat samples. DNA purity was measured based on absorbance at 260 and 280 nm (NanoDrop Spectrophotometer) and all samples had a 260/280 nm ratio of 1.8–2.0.

LTL measurement was carried out with an adaptation of the qPCR methods described by Cawthon (2002, 2009). The method is described and validated in more detail elsewhere (Bendix et al., 2014b).

For measurement of telomere repeat copies (T), primers Telg—5′ -ACACTAAGGTTTGGGTTTGGGTTT GGGTTTGGGTTAGTGT-3′ and Telc—5′-TGTTAGGTATCCC TATCCCTATCCCTATCCCTATCCCTAACA-3′ were used. The single copy gene (S) chosen was TaqMan Copy Number Reference Assay RNase P (Applied Biosystems Inc., CA, USA). The qPCR was performed on a 7900HT Fast Real-Time PCR System in 384-plates. Analyses were carried out using the 7900HT Sequence Detection System (SDS) version 2.3 (Applied Biosystems INC., CA, USA). Cycling conditions for telomere runs were 50°C for 2 min, 95°C for 2 min, followed by two cycles of 95°C for 15 s, 52°C for 15 s, and 36 cycles of 95°C for 15 s, 62°C for 15 s, and 71°C for 15 s. LTL was measured in T/S ratio.

LTL was measured twice in two independent batches (*n* = 95 group 1 and *n* = 99 group 2).

All the analyses presented in this paper were carried out separately on each of the two telomere measurements (12 results failed for one measurement and 4 results failed for the other measurement). As the results were nearly identical only data for one of the measurements is presented (*n* = 93 group 1 and *n* = 97 group 2).

### Covariables

A clinical neurocognitive test battery was used to assess functions proved to be sensitive to normal and pathological cognitive decline related to aging. For this study we included the Addenbrooke's (ACE) test (Mathuranath et al., 2000), a brief cognitive screening battery that is sensitive to mild cognitive impairment. As depressive symptoms might influence cognitive performance, data from the Major Depression Inventory (MDI), which measures the frequency of 12 depressive symptoms over the past 2 weeks (Bech et al., 2001) collected in the CAMB study, were included. The two mental scores were analyzed as continuous variables. In addition, covariables which have been associated with cognitive decline and LTL (information on vocational education, current smoking status, alcohol consumption, physical activity during leisure, BMI and serum cholesterol) were included in the analyses. These variables were obtained in the CAMB examination in 2009-10 (participants aged ~56 year).

### Statistical analysis

Differences in LTL and covariables between the two cognitive groups were analyzed by *t*-test and univariate linear regression models for categorical variables. We then conducted series of multiple regression analyses to evaluate the individual and mutually adjusted influences of the potentially confounding covariables (smoking, alcohol consumption, leisure time activity, BMI, serum cholesterol, MDI, ACE, and vocational education) on the association between the two groups with different change in cognitive function and LTL. In the first regression model we included smoking, alcohol, leisure time activity, BMI and cholesterol. Subsequently ACE and MDI were added while vocational education was also included in the final model. The basic assumption for linear regression [linearity, normality and colinarity (Variance Inflation Factor)] was examined using the diagnostic facilities in STATA.

## Results

Due to selection procedure, there was no difference between the two groups on BPP scores (measured ~20 years), but substantial differences on the IST scores (measured ~56 years). Group 2 also obtained lower ACE scores, had less education, higher cholesterol and higher MDI depression scores, while there was no difference between the groups with regard to prevalence of smoking, sedentary leisure time activity, alcohol consumption or BMI (Table 1).

**Table 1:** **Mean leukocyte telomere length (LTL) (age ~58) and the distribution of covariables in 190 middle-aged men with positive (Group 1) and negative (Group 2) cognitive change between age ~20 and 56 years**.

	**Positive cognitive change *N* = 93**	**Negative cognitive change *N* = 97**	*P*-value^*^	**LTL mean (SD)**	*P*-value^*^
LTL (mean (SD))	1.08 (0.28)	0.98 (0.24)	*P* < 0.01		
Percent smoking at age 56					
Yes	18.3	16.5		1.06 (0.31)	
No	81.7	83.5	*P* = 0.74	1.02 (0.25)	*P* = 0.48
Percent with weekly alcohol consumption above 21 drinks at age 56					
Yes	20.4	22.6		1.00 (0.25)	
No	79.6	77.3	*P* = 0.71	1.04 (0.26)	*P* = 0.16
Percent Lower vocational education at age 56					
Yes	25.8	48.5		1.00 (0.27)	
No	74.2	51.6	*P* < 0.01	1.05 (0.25)	*P* = 0.15
Percent sedentary leisure time activity at age 56					
Yes	6.6	8.3		1.02 (0.22)	
No	93.5	91.7	*P* = 0.65	1.03(0.26)	*P* = 0.88
Body Mass Index (kg/m^2^) at age 56 (mean (SD))	26.21 (3.63)	26.94 (3.40)	*P* = 0.08	H 1.03 (0.28)	
				L 1.04 (0.24)	*P* = 0.40
Serum cholesterol (mmol/l) at age 56 (mean (SD))	5.30 (0.85)	5.54 (0.91)	*P* = 0.03	H 1.04 (0.24)	
				L 1.02 (0.27)	*P* = 0.86
MDI Depression score at age 56 (mean (SD))	5.30 (4.75)	7.33 (7.74)	*P* = 0.01	H 1.06 (0.27)	
				L 0.99 (0.24)	*P* = 0.04
BPP test score at age 20 (mean (SD))	46.3 (9.8)	45.3 (8.2)	*P* = 0.43	H 1.02 (0.27)	
				L 1.05 (0.24)	*P* = 0.21
IST test score at age 56 (mean (SD))	42.7 (7.4)	21.1 (6.1)	*P* < 0.01	H 1.00 (0.23)	
				L 1.07 (0.29)	*P* = 0.05
ACE at age 58 (mean (SD))	95.82 (3.35)	92.93 (4.66)	*P* < 0.01	H 1.00 (0.23)	
				L 1.07 (0.29)	*P* = 0.04

* *From t- or chi-square test; H, highest 50%; L, lowest 50% of distribution of the covariables; BPP, Børge Priens Prøve; IST, Intelligenz-Struktur-Test 2000 R; MDI, Major Depression Inventory; ACE, Addenbrooks cognitive test*.

Mean LTL measured at age ~58 was shortest in group 2, i.e., in the participants with negative residuals reflecting relative cognitive decline (*P* < 0.01) (Table 1). The covariables were not or only moderately associated with LTL (Table 1, last column). In the regression model (Table 2) with LTL as outcome and group as main predictor participants with negative change in cognitive performance during adult life had shorter mean LTL than did participants with positive change in cognitive performance (unadjusted difference β = −0.09, 95% CI −0.16 to −0.02, *p* = 0.02). This association remained significant after adjusting for smoking, alcohol consumption, leisure time activity, BMI and cholesterol (adjusted difference β = −0.09, 95% CI −0.17 to −0.01, *p* = 0.02) and a *p*-value of 0.05 was obtained after adjusting for smoking, alcohol consumption, leisure time activity, BMI, cholesterol, MDI and ACE (β = −0.08, 95% CI −0.16 to −0.00, *p* = 0.05). Association was non-significant after adjusting for smoking, alcohol consumption, leisure time activity, BMI, cholesterol, current cognitive function, depression and education (adjusted difference β = −0.07, 95% CI −0.16 to −0.01, *p* = 0.08).

**Table 2:** **The relationship in beta-coefficient and 95% confidence intervals (CI) of negative cognitive change (Group 2) vs. positive cognitive change (Group 1) between age ~20 and 56 years with leukocyte telomere length (LTL) (age ~58) in 190 middle-aged Danish men**.

	**Beta-coefficient (95% CI)**	***P*-value**
Negative cognitive change (group 2 vs. 1)	−0.09 (−0.16 to −0.02)	*P* = 0.02
Negative cognitive change group, adjusted for smoking, alcohol consumption, BMI, leisure time activity and cholesterol	−0.09 (−0.17 to −0.01)	*P* = 0.02
Negative cognitive change group, adjusted for smoking, alcohol consumption, BMI, leisure time activity, cholesterol, MDI and ACE	−0.08 (−0.16 to −0.00)	*P* = 0.05
Negative cognitive change group, adjusted for smoking, alcohol consumption, BMI, leisure time activity, cholesterol, MDI, ACE and vocational education	−0.07 (−0.16 to −0.01)	*P* = 0.08

BMI, Body Mass Index; MDI, Major Depression Inventory; ACE, Addenbrooks cognitive test.

## Discussion

In the present study we investigated the association between age-related change in cognitive function and LTL. In a sample of middle-aged men selected on the basis of change in cognitive function between age ~20 and 56, LTL measured at age ~58 was significantly shorter in the group with negative change in cognitive function than it was in the group with a positive change. This relationship attenuated after adjustment for education, smoking, alcohol consumption, BMI, leisure time activity, serum cholesterol and scores for current cognitive function and depression. Repeated measurements of LTL were not available. Therefore, association of cognitive decline and LTL measured at different ages can't be examined or whether cognitive decline precede changes in LTL. Furthermore, the effect of the current cognitive function measured by the ACE test on LTL compared to the impact of cognitive changes over the life course on the telomere length can't be sufficiently concluded. These results do not concur with another study in the literature of ~1000 relatively healthy men and women from Scotland in which no relationship was found between LTL and changes in cognitive function over the life course (Harris et al., 2012). As noted by the authors of this study, their population was socially homogenous and the variance possibly too limited to detect any association. Two other cohort studies with shorter follow-up times found that short LTL predicted cognitive decline in old age (Devore et al., 2011; Yaffe et al., 2011), while a previous study on geriatric patients with a mean age of 85 did not find that low LTL predicted progression from normal to mild cognitive impairment after 2 years of follow-up (Zekry et al., 2010).

The direction of the association between cognition and telomere length is unclear. The association may be bi-directional or it may be caused by related underlying processes. As an example increasing obesity is negatively correlated with cognitive performance supported by both human clinical and animal studies but the mechanisms have not been fully clarified. Studies have suggested obesity to negatively impact cerebral vascular function, hippocampal brain atrophy, blood-brain barrier functions and inflammation contributing to cognitive deficits (Nguyen et al., 2014). Furthermore, telomere length has been suggested to be affected by obesity among other factors such as smoking, physical activity, and alcohol consumption (Mather et al., 2011). On the other hand, telomere length may impact pulse pressure and the risk of stroke which in turn may lead to cognitive impairment. An comprehensive causal explanation of these associations and the importance of each involved factor have not been fully clarified (Jeanclos et al., 2000; Waldstein et al., 2008; Cai et al., 2013; Eitan et al., 2014; D'Mello et al., 2015).

The strength of the present study is that it was based on prospectively collected data on cognitive performance in a sample of men with no clinical signs of mental disorder or dementia. To obtain sufficient variance, the two groups were selected to consist of men with extreme residuals, i.e., those with the most negative and positive relative change in cognitive function. In order to account for variation in LTL measurements, qPCR measurements were repeated in two independent batches. Results from the analyses based on each of the two measurements showed the same pattern of associations. The assessment of cognitive function was conducted with two different tests at age ~20 (BPP) and at age ~56 (IST), but with an almost 40 year retest interval a correlation of 0.70 has been observed between the two tests in the larger cohort. This would suggest that errors of measurement and differences between the two tests contribute relatively little variance and that the residuals reflect true change in global cognitive function (Osler et al., 2013). It would have been advantageous for the present study if longitudinal LTL data were available. Longitudinal LTL data would provide information whether individual short telomere length was innate or had in fact decreased during life due to for example inflammation and oxidative stress. Moreover, repeated measurements of LTL and of cognitive abilities across lifespan would provide valuable insight into the onset and rate of changes as well as strength of relationship between cognition and LTL at different stages of life.

Another limitation of the study is the measurement of telomere length in a heterogeneous blood sample and the used method doesn't discriminate between telomere lengths in different cell types or different chromosomes. It would have been advantageous to measure telomere length in neural tissue such as neurons or glial cells if materials like this were available. An additional benefit for the study could be to measure telomere length not just by one method to correct for PCR measurement uncertainty.

A recent study based on the Jerusalem Lipid Research Clinic study has examined longitudinal LTL data and association with cognitive ability (Cohen-Manheim et al., 2016). The LTL dynamics was measured over 13 years in young adults (measured at ages 28–32 and 41–46 years) and compared with cognitive function at ages 48–52. In this study increased LTL shortening was found to be associated with poorer cognitive function. However, another new study measuring telomere attrition and cognitive function at later ages (from 70 to 76 years and from 79 to 92 years) did not find the rate of telomere attrition to correlate with the rate of cognitive decline at these high ages (Harris et al., 2016). The rate of LTL changes may depend on different factors that vary across the life span and telomere length dynamics may differ at different stages in life. Therefore, it is likely that the association with age-related cognitive decline will change according to the timing of telomere analysis and age at cognitive assessment (Mather et al., 2011).

In conclusion, our study suggests that preclinical cognitive changes in middle-aged men are associated with LTL at age 58.

## Author contributions

LR: Design of the work, analysis and interpretation of data for the work. Revision of the work for intellectual content and according to referee suggestions. LB: Design of the work, analysis and interpretation of data for the work. Revision of the work for intellectual content. MH: Experimental work and analysis of data for the work. Revision of the work for intellectual content. BF: Design of the work and interpretation of data for the work. Revision of the work for intellectual content. EM: Conception and design of the work and interpretation of data for the work. Revision of the work for intellectual content. ML: Conception and supervision of the work. Revision of the work for intellectual content. MO: Conception and design of the work including statistical calculations, analysis and interpretation of data for the work. Drafting and revision of the work for intellectual content and according to referee suggestions.

### Conflict of interest statement

The authors declare that the research was conducted in the absence of any commercial or financial relationships that could be construed as a potential conflict of interest.
