# Supplementary material for: Cognitive Change during the Life Course and Leukocyte Telomere Length in Late Middle-Aged Men
Source: Front Aging Neurosci. 2016 Dec 9;8:300. doi: 10.3389/fnagi.2016.00300 (PMC5145851; doi:10.3389/fnagi.2016.00300)
Supplement: Supplementary file 1 [file Table1.PDF]

Supplementary Material

Cognitive Change During the Life Course and Leukocyte Telomere Length in Late Middle-aged Men.

PhD Lene Rask[1,2,8,*], PhD Laila Bendix[3], PhD Maria Harbo[4], PhD Birgitte Fagerlund[5], Professor Erik Lykke Mortensen[6,8], Professor Martin Lauritzen[1,2,8,*], Professor Merete Osler[6,7]

1. Department of Neuroscience and Pharmacology, University of Copenhagen, Copenhagen, Denmark
2. Department of Clinical Neurophysiology, Rigshospitalet - Glostrup, Glostrup, Denmark
3. Pain Research Group, Department of Anaestesiology and Intensive Care Medicine, Odense University Hospital, Odense, Denmark
4. Department of Clinical Genetics, Vejle Hospital, Vejle, Denmark
5. Center for Neuropsychiatric Schizophrenia Research and Lundbeck Foundation Centre for Clinical Intervention and Neuropsychiatric Schizophrenia Research, University of Copenhagen, Psychiatric Centre Glostrup, Glostrup, Denmark.
6. Department of Public Health, University of Copenhagen, Copenhagen, Denmark
7. Research Center for Prevention and Health, Glostrup Hospital, Glostrup, Denmark
8. Center for Healthy Aging, University of Copenhagen, Copenhagen, Denmark

Corresponding authors: Lene Rask and Martin Lauritzen, lene.rask.01@regionh.dk and martin.johannes.lauritzen@regionh.dk

Supplementary Table 1 Studies of leukocyte telomere length (LTL) and cognitive decline

Author, year of publication and [ref-number]	**Method for cognitive assessment**	**Cognitive status**	**Study population**	**Study design**	**Methods for LTL**	**Number**	**Age**	**Main findings**	Adjustments
Harris 2006 [1]	MMSE, MHT	Non-demented	Lothian Birth Cohort	Cross-sectional	TRF	190	79y	No association with LTL and cognitive decline. Small association to verbal fluency only	Education
Valdes 2010 [2]	CANTAB	Non-demented	TwinsUK cohort	Cross-sectional	TRF	382	19-78y ♀	LTL correlates with better cognitive performance	Age,SEP, BP,glucose, BMI, smoking, exercise, VitD,
Bendix 2011 [3]	MMSE, cognitive composite	78/548 MMSE<24	Danish twins	Cross-sectional	TRF	548	73-92y	No association between LTL and cognitive function	Age, gender and exercise
Der 2012 [4]	Alice Heim 4, reaction time (RT)		West of Scotland Community	Cross-sectional	qPCR	2185	36; 56, 76y	Associated to RT and AH4 score. However diminished after adjustment for age. Little to offer for prediction.	Age
Ma 2013 [5]	MMSE, community screening interview	Incl. MMSE<24	Chinese community-dwelling male	Cross-sectional	qPCR	976	>65y ♂	No linear association with LTL and MMSE. Shorter LTL in individuals with MMSE<24	Age, education

Martin-Ruiz 2006 [6]	MMSE	Non-demented	Stroke survivors	Cohort (2 y follow-up) LTL → Dementia	TRF	195	80±4y	Longer LTL associated with reduced risk of dementia and less reduction in MMSE	Age,BP,ApoE4, CVD
Yaffe 2009 [7]	MMSE,<br>DSST	Non-demented	Health ABC	Cohort (7 y follow up)<br>Cognitive decline → LTL	qPCR	2741	70-79y	Cognitive decline was less for longest tertile of LTL than medium and short for MMSE, not DSST	Age, gender, SEP
Mather 2010 [8]	Cognitive performance		Australian population	Cohort (4 y follow up) Cognitive decline → LTL	qPCR	646	40´s & 60´s	No cross-sectional association with LTL and cognitive function, nor to cognitive decline	Age, gender, smoking alcohol, exercise, BP
Devore 2011 [9]	TICS East Boston		Nurses Health study	Cohort (10y follow up)<br>LTL → Cognitive decline	qPCR	2092	~65y<br>♀	Longer LTL associated with slower cognitive decline. Not a powerful marker.	Age, education, smoking, DM, BP
Harris 2012 [10]	MH-T, speed, WAIS-III	Relatively healthy	Lothian Birth Cohort	Cohort (60y follow up) Cognitive decline → LTL	qPCR	1048	70y	No association to cognitive performance at age 70 nor to change from 11-70y	Gender, education, smoking, alcohol

MMSE=Mini Mental State Examination; MHT=Moray house test; CANTAB=Cambridge Neuropsychological Test Automated Battery; DSST=Digit symbol substitution test; TICS=Telephone interview for cognitive status; WAIS=Wechsler Adult Intelligence Scale; TRF=Telomere restriction fragment length assay; BP= Blood pressure; BMI= Body Mass Index; SEP socioeconomic Position; CVD= Cardiovascular disease; DM= Diabetes; Baseline exposure → Outcome at follow-up

References

1. Harris SE, Deary IJ, MacIntyre A, Lamb KJ, Radhakrishnan K, Starr JM et al. 2006 The association between telomere length, physical health, cognitive ageing and mortality in non-demented people. *Neuroscience Letters*; 406:260-4

2. Valdes AM, Deary IJ, Gardner J, Kimura M, Lu X, Spector TD et al. 2010 Leucocyte telomere length is associated with cognitive performance in healthy women. *Neurobiol Ageing*; 31:986-92

3. Bendix L, Gade MM, Staun PW, Kimura M, Jeune B, Hjelmborg JV et al. 2011 Leukocyte Telomere Length and Physical Ability among Danish Twins age 70+. *Mech Ageing Dev*. 132(11-12):568-72

4. Der G, Batty GD, Benzeval M, Deary IJ, Green MJ, McGlynn L et al. 2012 Is telomere length a biomarker for aging: cross-sectional evidence from the west of Scotland? *PLoS One*.;7:e45166

5. Ma SL, Lau ESS, Suen EW, Lam LC, Leung PC, Woo J et al. 2013 Telomere length and cognitive function in southern Chinese community-dwelling male elders. *Age & Ageing*; 42:450-5

6. Martin-Ruiz C, Dickinson HO, Keys B, Rowan E, Kenny RA, Von Zglinicki T et al. 2006 Telomere length predicts poststroke mortality, dementia, and cognitive decline. *Ann Neurol.*; 6:174-80

7. Yaffe K, Lindquist K, Kluse M, Cawthon R, Harris T, Hsueh WC et al. 2011 Telomere length and cognitive function in community-dvelling elders: Findings from the Health ABC study. *Neurobiology of ageing*; 32:2055-60

8. Mather KA, Jorm AF, Anstey KJ Milburn PJ, Easteal S, Christensen H. 2010 Cognitive performance and leukocyte telomere length in two narrow age cohorts: a population study. *BMC Geriatr.;* 10:62

9. Devore EE, Prescott J, De Vivo I, Grodstein F. 2011 Relative telomere length and cognitive decline in the Nurses' Health Study. *Neuroscience letter*; 492:15-8

10. Harris SE, Martin-Ruiz C, von Zglinicki T, Starr JM, Deary IJ. 2012 Telomere length and aging biomarkers in 70-year olds: the Lothian Birth Cohort 1936. *Neurobiol Aging*; 33:1486.e3-8
